# Pharmacological Antagonism of T-Type Calcium Channels Constrains Rebound Burst Firing in Two Distinct Subpopulations of GABA Neurons in the Rat Ventral Tegmental Area: Implications for α-Lipoic Acid

**DOI:** 10.3389/fphar.2019.01402

**Published:** 2019-11-26

**Authors:** Taylor Joel Woodward, Vesna Tesic, Tamara Timic Stamenic, Vesna Jevtovic-Todorovic, Slobodan M. Todorovic

**Affiliations:** ^1^Program in Neuroscience, Indiana University-Bloomington, Bloomington, IN, United States; ^2^Department of Psychological and Brain Sciences, Indiana University-Bloomington, Bloomington, IN, United States; ^3^Department of Anesthesiology, University of Colorado, Aurora, CO, United States; ^4^Neuroscience Graduate Program, University of Colorado, Aurora, CO, United States

**Keywords:** ventral tegmental area, gamma-amino-butyric acid, T-type calcium channel, lipoic acid, rebound spiking, burst firing, low-voltage-activated

## Abstract

The ventral tegmental area (VTA) is a midbrain region highly involved in motivation and reward. A large body of work has investigated synaptic plasticity and ion channel excitability in this area, which has strong implication in drug abuse. We recently provided electrophysiological and pharmacological evidence that the Ca_V_3.1 isoform of T-type voltage-gated calcium channels contributes to the excitability of VTA dopamine (DA) neurons. However, the role of T-channels in excitability of VTA gamma-amino-butyric acid (GABA) neurons remained unaddressed. Here, with a population study of rat VTA GABA neurons, we provide evidence that T-channels contribute to rebound spiking activity in two phenotypically distinct subpopulations of GABAergic neurons, each with differing electrophysiological characteristics. Additionally, we provide the first study to investigate the effect of α-lipoic acid (ALA) on ion channels in mesolimbic reward circuitry. Taken together, our population study and pharmacology experiments implicate T-channels as a target for therapies aimed at tempering VTA and mesolimbic circuit excitability.

## Introduction

The ventral tegmental area (VTA), which provides strong dopaminergic projections to the ventral striatum, prefrontal cortex, amygdala, and hippocampus, plays a vital role in motivation and reward processes. The VTA, consisting of dopamine (DA) neurons (∼65%), gamma-amino-butyric acid (GABA) neurons (∼30%) and glutamatergic neurons (∼5%) (Yamaguchi et al., 2007; Nair-Roberts et al., 2008), has been implicated in many neurobiological and neuropathological functions including substance abuse, arousal (Yu et al., 2019), pain modulation (Jarcho et al., 2012; Navratilova et al., 2015), and affective states (Nestler and Carlezon, 2006). These phenomena have sparked interest into the mechanisms of neural plasticity in the VTA and the respective roles of various ionotropic and metabotropic receptors that contribute to neuronal excitability, plasticity, and circuit function.

Changes in neuronal excitability can drastically affect how a neuronal network operates and transmits information. Various studies have investigated excitability of both DA and GABA subpopulations of VTA neurons as mechanisms for drug seeking behaviors, and electrophysiology studies have shown that their excitability are affected during different stages of the drug seeking cycle (Kanunnikova and Vinitskaya, 1997; Nelson et al., 2018). Dopaminergic transmission in mesolimbic structures is highly implicated in reward, and results from increased activity of VTA DA neurons, which subsequently release DA into ventral striatum structures in larger and more frequent volleys. Importantly, it has been proposed that hyperexcitability of VTA GABA neurons contribute to negative affective state during withdrawal (Nestler and Carlezon, 2006; Vargas-Perez et al., 2014). Others have demonstrated alterations in GABAergic transmission and increased GABA-release in the VTA and nucleus accumbens (NAc) in animals during withdrawal from opioids and ethanol (Bonci and Williams, 1997; Chieng and Williams, 1998; Ibrahim et al., 2000; Kaufling and Aston-Jones, 2015; Nelson et al., 2018).

T-type calcium channels (T-channels) are low-voltage-activated (LVA) calcium channels that have been extensively studied in the thalamus and allow for burst firing from near-resting membrane potential states (Stamenic and Todorovic, 2018). As they recover from inactivation at around -80 mV, a small hyperpolarizing current can drive a calcium influx that allows for rapid burst firing from lower membrane potentials in neurons containing T-channels. Recently, our lab investigated the role of the Ca_V_3.1 isoform of T-channels in VTA neuronal excitability and confirmed the presence of T-channels on both DA and non-DA neurons using a tyrosine-hydroxylase (TH) Tg-GFP rat and mouse genetics with Ca_V_3.1 knockout mice (Tracy et al., 2018). Interestingly, we found that while some DA neurons expressed T-channels, it was actually likely that T-channel dependent rebound bursting was somewhat more prominently expressed in non-DA neurons. Here, we continue our study by employing the ability of the VGAT Venus rat line (Uematsu et al., 2008) to preferentially investigate GABAergic neurons and provide functional evidence showing that the majority of GABA neurons in the VTA are likely to express T-channels and show a typical post-inhibitory rebound firing pattern.

Additionally, we explored the hypothesis that pharmacological modification of T-channels could be a potential treatment of disorders involving the VTA. We specifically tested whether the antioxidant α-lipoic acid (ALA), which previously was shown to block T-channels in dissociated dorsal root ganglion, as well as recombinant Ca_V_3.1 and Ca_V_3.2 isoforms of T-channels (Lee et al., 2009), could affect excitability in a brain slice preparation. We found acute ALA application decreased post-inhibitory rebound spiking, characteristic of T-channel activity, as well as tonic firing induced by membrane depolarization. In summary, this study shows that VTA GABA neurons exhibit functionally distinct populations in terms of T-channel activity, and that ALA can inhibit their different firing modes. Thus, naturally occurring therapeutic agents that affect T-channels may have the ability to alter excitability/firing modality of sub-populations of neurons in the VTA while sparing others.

## Materials and Methods

### Animal Care

VGAT Venus Tg(+) rats (RGD Cat# 2314363, RRID: RGD_2314363) were housed in compliance with IACUC protocols on a 12 h light/dark cycle. VGAT Venus (+) rats were bred with a single wild type Wistar mate. Pups were tail snipped for genotyping between postnatal day 5 and 8, and weaned between postnatal day (P)21 and P28. All animals used for immunofluorescence and electrophysiology were between P16 and P26. All procedures were approved by the Institutional Animal Care and Use Committee of the University of Colorado-Denver, and were carried out in accordance with NIH guidelines.

### Confocal Laser Scanning Microscopy

Transgenic VGAT Venus rats were deeply anesthetized with 5% isoflurane and transcardially perfused with phosphate buffered saline (PBS pH7.4, Life Technologies), followed by 4% paraformaldehyde in 0.1 M phosphate buffer, pH 7.4 (PFA). Whole brains were extracted and post-fixed in PFA for 24 h. Brains were rinsed in PBS, embedded in 3% agarose and brain sections (50 µm) were prepared on a microtome (Leica VT1200). Slices were rinsed three times in PBS, mounted on slides and heat antigen retrieval process was performed in boiling citric buffer (0.1M, pH6.0). Sections were permeabilized in 1% glycine in PBST for 15 min and rinsed in PBS for 5 min. Sections were then blocked with 5% normal donkey serum in PBST (0.1% Triton X 100 in PBS) for 30 min, and incubated with primary antibodies (chicken anti-NeuN, 1:250, Millipore, goat anti-Venus, 1:250, MyBioSource) diluted in 1% normal donkey serum in PBST overnight at 4°C. Slices were rinsed 3 × 5 min in PBST followed by 5 min rinsing in PBS and then incubated for 2 h with secondary anti-rabbit antibody (anti-chicken Alexa 647 and anti-goat Alexa 488, 1:500 for both, Invitrogen) at room temperature and washed 3 × 5 min in PBS. The sections were mounted in fluorescent mounting medium (Vector laboratories) and the fluorescent signals were obtained using an Olympus FluoView FV1200 confocal laser scanning microscope at 20× magnification. Specificity of the antibody was confirmed by omitting the primary antibody in control slices.

### *In-Vitro* Whole Cell Electrophysiology

#### Slice Preparation

VGAT-Venus (+) pups were sacrificed for electrophysiology recordings from postnatal day 16 to 26 for all recordings. VTA GABA neurons were recorded from a total of 21 male and female transgenic rats from 7 separate litters.

Pups were deeply anesthetized with isoflurane and decapitated. Their brains were rapidly extracted and placed in a prechilled solution containing in mM, as follows: sucrose 260, D-glucose 10, NaHCO3 26, NaH2PO4 1.25, KCl 3, CaCl2 2, and MgCl2 2. Brains were glued ventral side-up to a pedestal and 250 µm horizontal sections containing the VTA were cut with a Leica VT 1200S vibratome (Leica Biosystems, Buffalo Grove, IL). Slices were placed in a 37°C solution containing, in mM: NaCl 124, D-glucose 10, NaHCO3 26, NaH2PO4 1.25, KCl 4, CaCl2 2, and MgCl2 2. Slices were incubated for 30 min and then equilibrated to room temperature, at which electrophysiology experiments were performed. We also examined the effects of more physiological temperatures on the excitability of VTA neurons in a limited number of experiments, and these results are presented in the Supplemental Material (**Supplemental Figure S1**).

VTA GABA neurons were visualized with an Olympus BX51WI microscope. All recordings presented were taken from VGAT-Venus (+) neurons, which were confirmed by briefly illuminating the slice with a blue light and viewing through a filter. After each recording, cell location was marked on an anatomical diagram using traditional landmarks (see results), and if there was doubt about whether the neuron was within the boundaries of the VTA, it was excluded from analysis. Neuronal membrane potentials were recorded in current clamp mode, amplified with a Multiclamp 200B amplifier (Molecular Devices, Foster City, CA, USA), and digitized with a Digidata 1440A (Molecular Devices). All signals were filtered at 2 kHz. Microelectrodes were fabricated from borosilicate glass with a filament and outer diameter of 1.2 mm using a P-1000 pipette puller (Sutter Instruments). Initial pipette resistance was between 3.5 and 6 MΩ. Current command protocols were carried out and recorded using Clampex 8.3 Software (Molecular Devices). Resulting voltage traces were analyzed using Clampfit 10.5 (Molecular Devices).

#### Current Clamp

Current clamp recordings were taken in the presence of 20 µM picrotoxin, 10 µM 2,3-dihydroxy-6-nitro-7-sulfamoyl- benzo[f]quinoxaline (NBQX) and 50 µM DL-2-amino-5-phosphonopentanoic acid (AP-V) in the extracellular medium, which consisted of the following (in mM): NaCl 125, d-glucose 25, NaHCO3 25, NaH2PO4 1.25, KCl 2.5, MgCl2 1, and CaCl2 2. The internal solution used in the pipette was potassium-D-gluconate 130, EGTA 5, NaCl 4, CaCl2 0.5, HEPES 10, Mg ATP 2, Tris GTP 0.5, and pH 7.2. Extracellular medium was bubbled constantly with 95% O_2_ and 5% CO_2_ and pH was assessed regularly to ensure no drastic alterations in pH during recording or pharmacology.

Cells were excluded from analysis if resting membrane potential was more depolarized than −45 mV or if their resting membrane potential varied by more than 5 mV during recording. Resting membrane potential for each cell was recorded and then cells were injected with small amounts of current (if necessary) to bring them to around −60 mV for excitability studies. Our “dual step” protocol (see **Figure 2A** and **Figure 5D**) altered membrane voltage with stepwise positive and negative current injections of 50 pA each sweep. A separate input-ouput protocol was used to more accurately assess tonic firing, which consisted of 25 pA increasing steps of current after an initial 100 pA injection. Hyperpolarization-induced current (Ih) was determined by the presence of a downward voltage sag at the beginning of the hyperpolarizing current injection greater than 3 mV in difference from the end of the pulse. If a cell showed less than 3 mV of difference between the end of the hyperpolarizing pulse and the antipeak of the pulse, it was classified as Ih negative.

### Pharmacology

Only cells that displayed stable patch characteristics were used for pharmacology experiments. Baseline recording were taken for 10 min, after which drug was superfused. Due to limited permeability of drug superfusate in the slice preparation, actual concentrations may be lower than reported. Cells were allowed at least 10 min incubation with drug for pharmacology experiments.

### Drugs

A stock of α-lipoic acid (purchased from Sigma-Aldrich, St. Louis, MO) was dissolved at a concentration of 1M in 100% ethanol (EtOH) and stored at 4°C at a concentration of 1M and diluted in extracellular medium to a concentration of 1 mM for pharmacology experiments. 3,5-dichloro-N-[1-(2,2-dimethyltetrahydro-pyran-4-ylmethyl)-4-fluoro-piperidin-4-ylmethyl]- benzamide (TTA-P2, from Alomone Labs, Isreal) was dissolved at a concentration of 5 mM in 100% di-methyl-sulfoxide (DMSO) and stored in aliquots at −20°C. Final concentrations of EtOH and DMSO were both.1% by volume for experiments. NBQX, AP-V, picrotoxin and all reagents/buffers in intra-pipette solutions were purchased from Sigma-Aldrich (St. Louis, MO).

### Statistics

pClamp Software version 10.0 (Molecular Devices, RRID : SCR_011323) was used to analyze electrophysiology recording characteristics. Statistical analyses were performed using Prism 7.0 (GraphPad Software RRID : SCR_002798), and figures were generated with Prism 7.0 or Origin 2018 (OriginLab).

Population studies were analyzed using either an unpaired parametric t-test or a One-Way Analysis of Variance (ANOVA) with a Fisher’s Least Significant Difference (LSD) *post hoc* test where appropriate. Pharmacology experiments were analyzed with a one sample t-test (hypothetical value of 1), and all results are displayed numerically and graphically as the mean ± SEM. Significance in graphs is notated with * (p < .05), ** (p < .01), and ***(p < .001).

## Results

Our recent patch-clamp recordings in acute VTA brain slices from WT rats clearly demonstrated expression of classic T-type currents that underlie post-inhibitory rebound burst firing in a subset of VTA neurons (Tracy et al., 2018). However, it is well known that the VTA contains heterogeneous populations of dopaminergic neurons, as well as both inhibitory and excitatory interneurons, all of which play critical roles in regulating motivated behaviors (Morales and Margolis, 2017). Hence, we aimed to determine if there was a functional significance to our findings and if T-channels may support excitability in one of the both cell populations in VTA. To address this issue and identify GABAergic neurons in the VTA, we took advantage of transgenic rats with the VGAT Venus rat line (Uematsu et al., 2008) to preferentially investigate GABAergic neurons and to test the hypothesis that the majority of GABA neurons in the VTA may show a typical T-channel dependent post-inhibitory rebound firing pattern.

**Figure 1:**
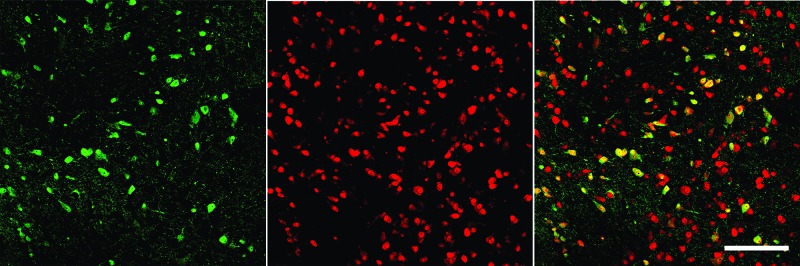
Immunohistochemistry of VTA neurons in VGAT-Venus rats. Double immunofluorescenct labeling of VTA neurons shows VGAT Venus-positive cells (green, left), NeuN positive cells (red, center) and an overlay showing the overlap (right). As not all neurons are VGAT+, we provide evidence that we were able to selectively identify and record from GABAergic neurons. Calibration bar on the right panel denotes 150 µm.

We employed confocal microscopy using brain slices from VGAT Venus rats (**Figure 1**) to examine the presence of GABA and non-GABA neurons. The general nuclear neuronal marker NeuN (red) was used to label all neuronal nuclei (both GABA and non-GABA neurons) in the VTA and adjacent areas in this region. A representative brain slice of the VTA region examined by dual imaging (**Figure 1**) shows VGAT-positive neurons (green color, left panel) and all neuronal nuclei labeled with NeuN (red color, middle panel). The right panel of **Figure 1** shows the merged image (yellow color). Similar images were obtained from a total of four VGAT Venus rats (2 males and 2 females), suggesting the VTA contains as expected, both GABA (Venus positive/NeuN positive) and non-GABA (Venus negative/NeuN positive) neurons, most of which are presumably DA neurons.

**Figure 2:**
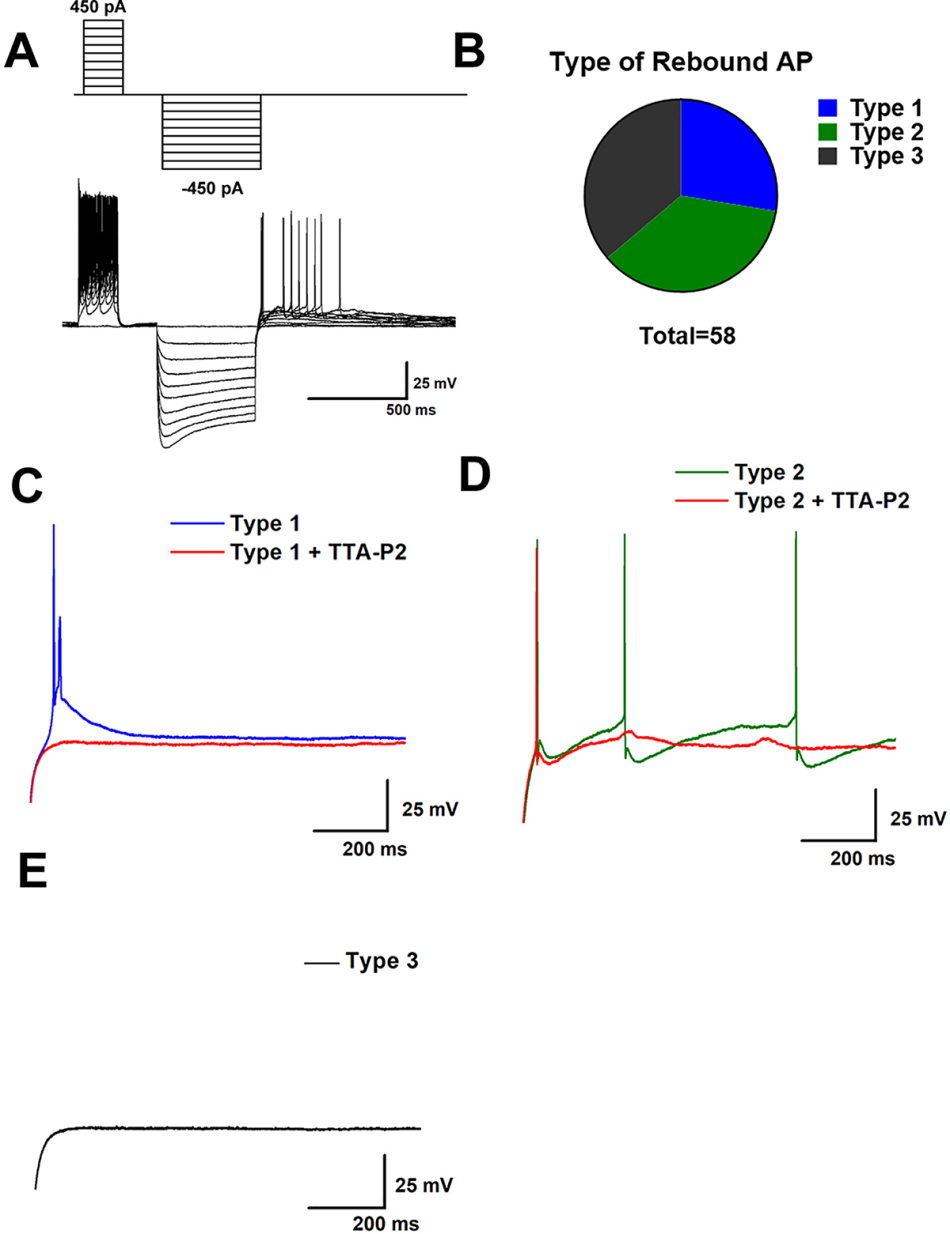
Sub-populations of VTA GABA neurons exhibit qualitatively distinct burst firing activity after membrane hyperpolarization. In **(A)** we show our current clamp protocol (above) and a corresponding neuronal membrane output (below) that allows for investigation of both tonic firing from increases in membrane voltage and burst firing after membrane hyperpolarization. A population study of 58 VTA GABA neurons found 3 phenotypically distinct firing patterns after injection of a hyperpolarizing current, as show in pie chart **(B)**. We categorized these observed phenotypes as Type 1, Type 2, or Type 3 based on their response to a hyperpolarizing current of 500 ms. Type 1 neurons as shown in panel **(C)** displayed fairly typical T-channel activity, marked by a classical depolarizing potential lasting 100–200 ms which included the firing of 1 or more action potentials during the slow depolarization. This low threshold calcium spike was blocked by 5 µM TTA-P2 Type 2 neurons, as shown in **(D)** also exhibited spiking after hyperpolarization. However, instead of a classical T-channel depolarization they generally exhibited fast membrane hyperpolarization following action potentials and a longer duration of spontaneous action potentials after hyperpolarizing pulses. Type 3 neurons, shown in **(E)**, were completely silent after hyperpolarization.

We next began our electrophysiological study with a functional population study of GABAergic neurons in the VTA to test the hypothesis that T-channels contribute to the excitability of these cells. We recorded from a total of 58 VGAT+ neurons from the VTA, identified in various VTA subregions (including caudal, rostral, medial, and lateral). In current clamp mode we used a dual-step protocol that injects positive current, followed by negative current in increasing steps (see **Figure 2A**). We found that after a hyperpolarizing pulse, membrane responses of VGAT+ neurons could be sorted into 3 categories, which we will refer to as Type 1, 2 and 3 neurons. The categories which we used are based on their response to a hyperpolarizing current injection, which can activate voltage gated ion channels and elicit a “rebound” action potential. Two distinct phenotypes of rebound spiking were observed. The first contains a classical low threshold calcium spike (LTCS), characterized by a post-hyperpolarization increase in membrane potential lasting 100–200 ms during which one or more action potentials may be fired (see **Figure 2C**). We found that applications of a selective T-channel antagonist 3,5-dichloro-N-[1-(2,2-dimethyltetrahydro-pyran-4-ylmethyl)-4-fluoro-piperidin-4-ylmethyl]- benzamide (TTA-P2) at a 5 µM concentration completely abolished LTCS and rebound action potentials in all Type 1 neurons tested pharmacologically. Type 2 neurons also exhibited one or more rebound action potentials, but instead of LTCS they showed a rapid membrane hyperpolarization after each action potential. **Figure 2D** shows a Type 2 neuron’s post-hyperpolarizing action potential and shows that TTA-P2 decreased, but did not completely abolish rebound firing in these neurons, suggesting that T-channels are important, but are not solely responsible for rebound bursting in these neurons. Type 3 neurons, as seen in **Figure 2E**, remained silent after a hyperpolarizing pulse. Pie chart **Figure 2B** shows similar proportions of Type 1, 2, and 3 neurons (16, 21, and 21 out of 58, respectively) that we identified in our study. Neurons from both males and females fell into these three categories in close to equal proportions. Age did not appear to be a significant factor in rebound phenotype, as all groups contained neurons from animals between 16 and 26 days old, with average group ages of 22.12 ± 0.94, 20.14 ± 0.70, and 19 ± 0.44 days for Type 1, 2, and 3 neurons, respectively.

**Figure 3:**
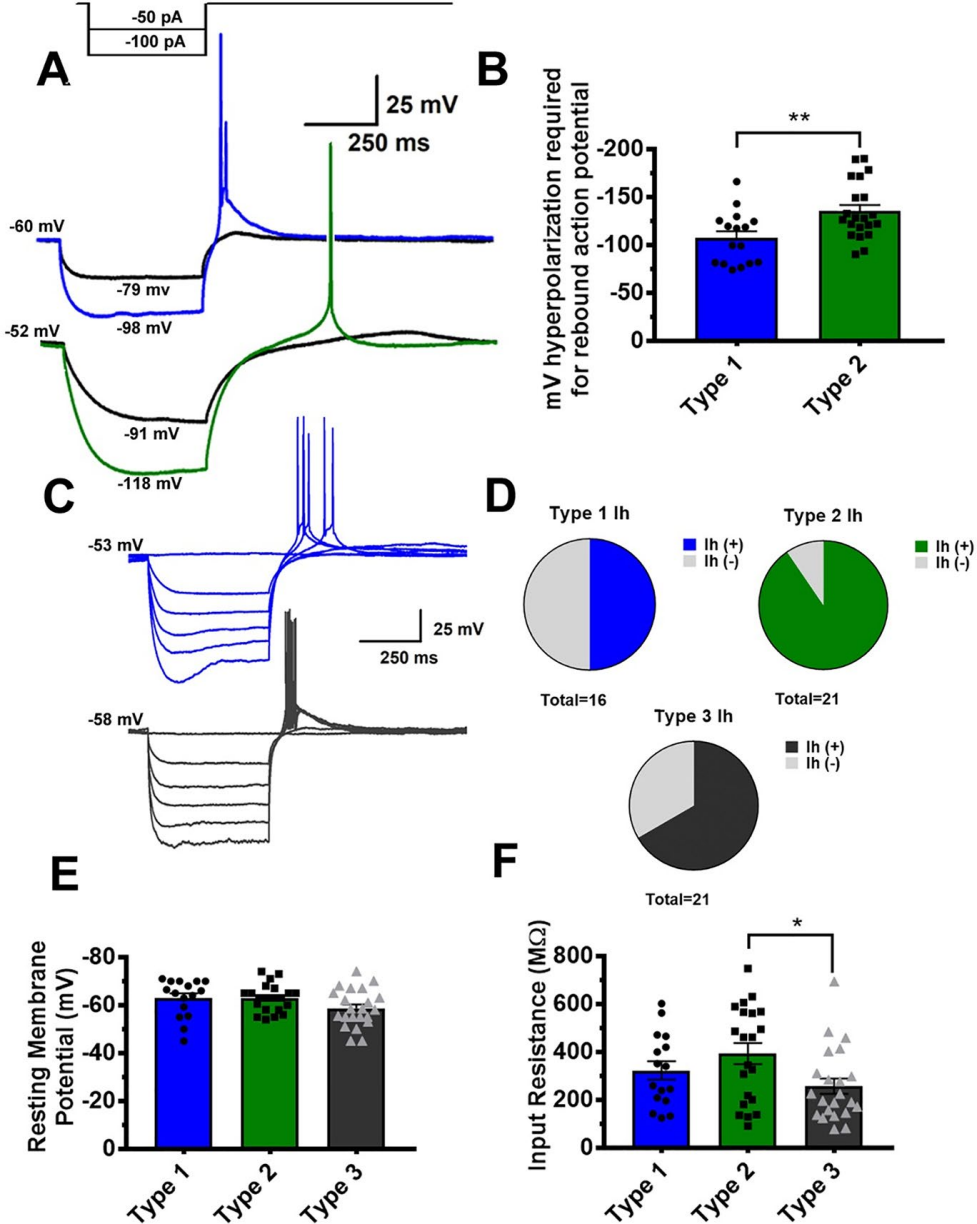
Quantitative analysis reveals Type 1 and Type 2 GABA neurons to require different levels of hyperpolarization to elicit a rebound action potential. A representative partial current injection protocol, shown in **(A)**, illustrates differences in Type 1 (blue) and Type 2 GABA neurons (green) **p = .0058. As shown in graph **(B)**, Type 2 neurons generally require a more hyperpolarized membrane potential to elicit a post-hyperpolarization “rebound” action potential. In **(C)** we show the presence of a voltage sag indicative of an Ih current at low potential in Type 1 neurons. A population analysis of all 3 GABA neuron classes reveals heterogeneity in Ih presence among the populations, although Type 2 neurons almost ubiquitously exhibited an Ih current **(D)**. It is of note that while resting membrane potential was even between groups **(E)**, Type 3 neurons had a slightly smaller average input resistance than Type 2 neurons, as shown graphically in *p = .0119 for graph **(F)**.

Quantitative analysis of VTA GABA rebound spiking revealed interesting differences between Type 1 and Type 2 neurons. Representative traces from Type 1 and Type 2 neurons in **Figure 3A** show that a lower membrane potential is required to evoke a rebound spike in Type 2 neurons. **Figure 3B** depicts the minimum membrane potential during the first hyperpolarizing current injection that elicited a rebound action potential in both groups. Additionally, we found heterogeneity in the presence of a hyperpolarization-induced voltage sag, indicative of Ih current. **Figure 3C** shows representative traces from 2 separate Type 1 VTA GABA neurons. The trace in blue (above) shows a distinct voltage sag, while the trace in grey (below) does not. We found this heterogeneity among all 3 populations, though it is of interest that Type 2 neurons almost ubiquitously expressed a hyperpolarization induced voltage sag, as seen in pie chart **Figure 2D**. Overall, resting membrane potential was not significantly different between groups, and no large differences in input resistance were noted between groups. The one exception is that a one-way ANOVA with multiple comparisons revealed Type 3 neurons to have a somewhat lower input resistance than type 2 neurons (see **Figure 3F**).

**Figure 4:**
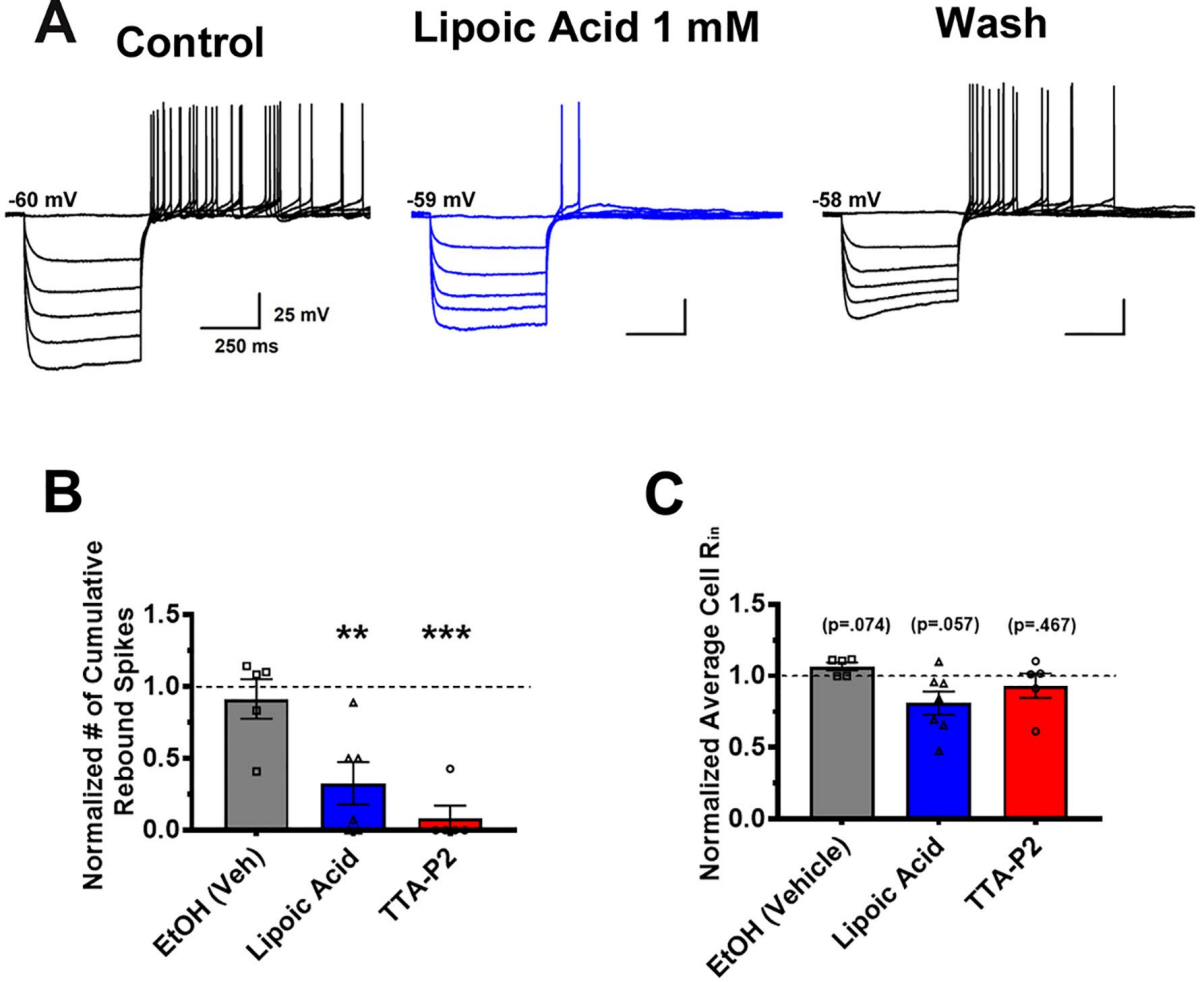
Application of lipoic acid reduces rebound action potentials in VTA GABA neurons. Representative trace **(A)** shows a clear, reversible decrease in rebound burst spiking after bath application of 1 mM ALA. As ALA was dissolved in EtOH as a vehicle, we bath applied 17 mM EtOH (vehicle), as well as EtOH vehicle + ALA to ensure results were not due to the effects of EtOH. As shown quantitatively in **(B)**, **p = .0061 normalization of cumulative action potentials per cell reveals that EtOH did not affect rebound spiking activity, while ALA significantly reduced post-hyperpolarization firing. A separate group of cells was treated with 5 µM TTA-P2, which completely eliminated rebound firing in all but 1 cell, ***p = .0004 to show the effects of a selective T-channel antagonist on VTA GABA neurons. Panel **(C)** reveals no changes in average input resistance during application of drugs between all groups.

Finally, we tested the hypothesis that pharmacological manipulation of T-channels could affect excitability of VTA GABA neurons by applying TTA-P2 and ALA to the extracellular buffer solution. As seen previously in representative traces 2C and 2D, TTA-P2 drastically reduced rebound spike count. As ALA was previously shown to inhibit T-channels in dorsal root ganglion, we hypothesized that it would be able to act on central nervous system ion channels as well. By comparing the cumulative number of recorded rebound spikes before and after drug superfusion, we determined that ALA reduced rebound firing as seen in representative trace **Figure 4A** and bar **Figure 4B**. As ALA was dissolved in 100% ethanol (EtOH) prior to dilution in the extra cellular medium, we wanted to test our vehicle for ALA as well, which after dilution was about 17 mM EtOH. We observed no change in the amount of rebound spiking after superfusion of 17 mM EtOH, shown in **Figure 4B**. **Figure 4C** shows that no drug application significantly impacted membrane input resistance, eliminating the potential confound that equal hyperpolarizing current injections would create significantly different membrane potentials. ALA was able to decrease rebound firing in both Type 1 and Type 2 neurons, though the majority of ALA neurons tested were Type 1.

**Figure 5:**
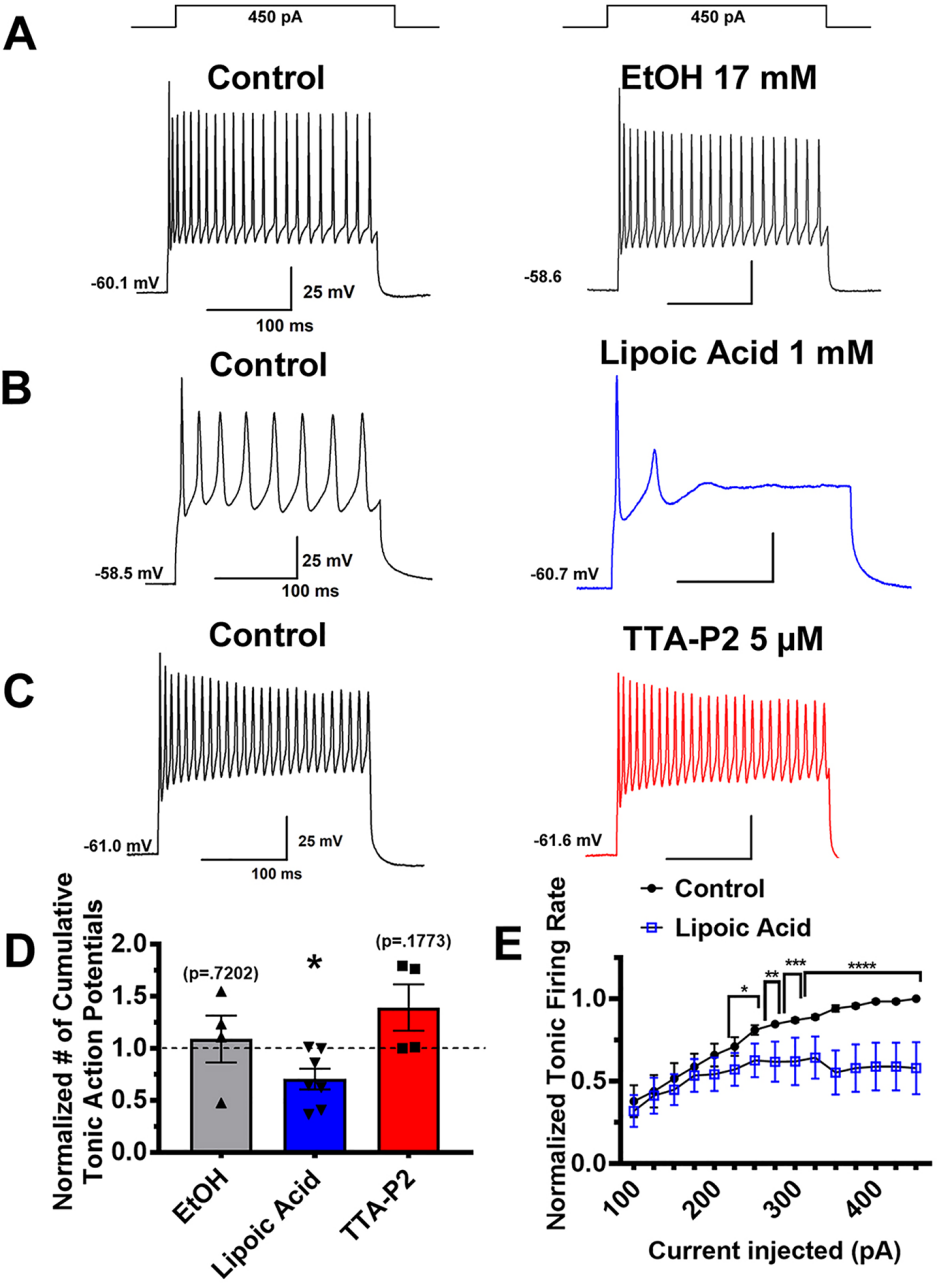
Different effects of T-channel inhibitors on tonic firing activity in VTA GABA neurons. Panels **(A**–**C)** on the left show representative neuronal membrane responses to stepwise depolarizing current injections in the control (pre-drug) conditions while the right side shows effect of different agents. The vehicle (ethanol) did not significantly affect tonic firing **(**panel **A)**. One mM ALA significantly inhibited tonic firing rate, represented by traces depicted in panel **(B)**. In contrast, superfusion of 5 µM TTA-P2 did not significantly alter the number of action potentials fired over the course of the stepwise injection **(**Panel **C)**. Bar graph **(D)** shows quantitative results of pharmacology experiments on tonic firing as the cumulative number of action potentials generated by the input-output protocol of neurons after application of drug compared to pre-treatment *p = .025. Panel **(E)** shows a more detailed effect of ALA on all current injection steps of the protocol *p < .05, **p < .01, *** p < .001, ****p < .0001.

To examine tonic firing, we used a stepwise depolarization protocol that yields an input-output curve of action potentials. While we previously saw TTA-P2 and ALA robustly reduce rebound firing, tonic firing mode was reduced to a lesser degree. As seen in representative traces **Figures 5A and C**, neither EtOH nor TTA-P2 significantly affected tonic action potential frequency. However, as shown qualitatively in representative trace **Figure 5B** and qualitatively in **Figure 5D**, ALA effectively decreased tonic firing activity evoked with stronger depolarizations. **Figure 5D** represents the normalized cumulative number of spikes generated by the input-output protocol, comparing post-treatment to pre-treatment. A complementary input-output curve for ALA presented in **Figure 5E** demonstrates that this effect occurred more robustly at stronger current injection steps. Together, these pharmacological studies show that ALA and TTA-P2 can effectively inhibit rebound burst firing, and that ALA also inhibits tonic firing in VTA GABA neurons.

## Discussion

In this study we determined that VTA GABA neurons exhibit functionally distinct populations in terms of T-channel activity, and that ALA can alter ion-channel specific activity in these populations.

Understanding the functionality of a system’s individual components can provide useful mechanistic insights into a system’s overall functionality. As the mesolimbic system has important implications in biological, psychological, and social issues, a great deal of neuroscientific research has looked into the mechanisms of this circuit on every level from molecular to systems analysis. Our previous finding that T-channels are present and functional in DA and non-DAergic VTA neurons opened the possibility of more molecular targets for drug therapies and better mechanistic understanding of VTA neuron activity. The study presented here provides valuable information about nuances when considering T-type calcium channels’ involvement in reward and motivation.

First, we give clear evidence that T-channels contribute to excitability of GABAergic neurons in the VTA through our use of the VGAT-Venus rat line. This transgenic rat provides the ability to visualize GABAergic neurons in the rat brain, while circumventing issues of altered GABA functionality seen in GAD67 Tg+ animals (Uematsu et al., 2008). Additionally, this rat line allows for easy translation of mechanistic studies to behavioral paradigms, perhaps through imaging techniques that could allow for assessment of neuronal activity during behavioral paradigms. As direct application of TTA-P2 abolished rebound spiking in all GABAergic neurons tested, we provide evidence that manipulations of T-channels can affect not only VTA DA excitability, but could be used to manipulate other aspects of VTA circuitry as well. Of interest is that in our previous study, TTA-P2 altered membrane resistance of DA neurons, where presently we found no such alteration in VTA GABA neurons. As functionally distinct populations of VTA GABA neurons were identified, it follows that agents that selectively manipulate T-channels could affect distinct neuronal populations and subpopulation in the VTA, while sparing others. This concept is also important to remember in the larger investigation of mesolimbic circuitry, as T-channels were recently found in other limbic structures such as the lateral habenula (Song et al., 2018) which is heavily implicated in aversion. Of note is our finding that, consistent with our previous investigation of VTA DA neurons, pharmacological blockade of T-channels by TTA-P2 in the VTA blocks rebound burst firing while leaving tonic firing unaffected.

In the earlier stages of studies involving electrophysiological recordings of VTA neurons, the Ih current was used as a cardinal marker of DAergic neurons. However, various groups have challenged this dogma, indicating the presence of an Ih current in GABAergic neuronal populations as well (for a review, see Margolis et al., 2006). Here, we confirm that VTA GABAergic neurons heterogeneously display a voltage sag characteristic of an Ih current at hyperpolarizing potentials, though it is worth noting that among our sample population, Type 2 neurons almost ubiquitously showed a voltage sag upon hyperpolarization. It is also worth noting that for practical reasons all recordings in this and our previuos study (Tracy et al., 2018) were done at room temperature. However, previous studies reported strong temperature dependence of both Ih and T-channel activation (Vargas and Lucero, 1999; Iftinca et al., 2006). Hence, it is likely that VTA cells recorded at physiological temperatures may be more excitable and likely to exhibit more prominent post-inhibitory rebound firing. Our preliminary experiments (**Supplemental Figure S1**) support this notion and this remains an important area of investigation for our future studies.

Our study also offers potentially useful insight into the field of the neurobiological underpinning of alcohol reward. As ALA was dissolved in a stock solution of 100% EtOH, we wanted to ensure that our vehicle was not the cause of the reduction of post-hyperpolarization rebound action potentials observed through application of ALA. Thus, we assessed responses to a 17 mM EtOH challenge (equal to the final concentration in which ALA was dissolved), seeing no alteration in post-hyperpolarization action potential activity. Hence, it appears that we can dismiss T-channels as a direct mediator of low dose ethanol reward. While other studies found similar conentrations of EtOH to block rebound firing in the thalamus (Mu et al., 2003), we found no change in rebound spiking of VTA GABA neurons.

α-lipoic acid (ALA) is a dietary supplement and powerful anti-oxidant that has been investigated preclinically for its abilities to attenuate various neuropathologies associated with metabolism and oxidative stress, such as cognitive decline with aging (Stoll et al., 1993; Farr et al., 2012; Zhang et al., 2019), various neuropathies (Yorek et al., 2017; Hidaka et al., 2019; Sun et al., 2019), and anesthetic neurotoxicity (Zhao et al., 2018). Promisingly, human clinical trials have shown ALA’s effectiveness in treating diabetic neuropathy, schizophrenia, and other pain disorders(Pessoa et al., 2015; Boriani et al., 2017; Sanders et al., 2017; Agathos et al., 2018). Previously, we showed ALA’s ability to reduce neuronal excitability of acutely dissociated dorsal root ganglion and partially inhibit recombinant Ca_V_3.1 and Ca_V_3.2 currents (Lee et al., 2009). The current study is, to our knowledge, the first investigation of ALA’s ability to alter specific ion channel activity in relatively intact central nervous system tissue.

The ability of ALA to reduce neuronal excitability and decrease rebound bursting is of special interest in light of various studies that have investigated it for addicion-related pathologies. Various groups showed that, in addition to protecting against intestinal damage from ethanol intake (Şehirli et al., 2008; Li et al., 2016), ALA can block ethanol reward (Ledesma and Aragon, 2013; Melis et al., 2015), decrease voluntary ethanol consumption in rats (Peana et al., 2013), and reduce ethanol-induced locomotor stimulation in mice (Ledesma and Aragon, 2012). As VTA GABA neurons become hyperexcitable during withdrawal from ethanol (Nelson et al., 2018), ALA could be potentially useful in tempering this phenomenon, which is thought to be associated with anhedonia and affective disturbance. While it has not been exhaustively studied in other preclincial behavioral paradigms relevant to addiction, ALA has also been shown to decrease tolerance and dependence from chronic morphine administration in rodents (Abdel-Zaher et al., 2013). Hyperexcitabilty of VTA GABA neurons also has been implicated in negative affect during withdrawal from opioids (Vargas-Perez et al., 2014). Understanding that ALA has the potential to attenuate both somatic dependence and psychological withdrawal from opioids, as well as treat various forms of pain shows promise in the search for alternative and supplemental pain treatments. We posit that ALA could be more thoroughly investigated as a companion/replacement of opioid analgesics.

The finding that ALA affects tonic firing from a membrane potential at which most T channels are inactive also indicates that ALA may act on other ion channels or proteins that affect excitability. An earlier *in vitro* study reported that ALA may also inhibit N-methyl-D-Aspartate (NMDA)-mediated ionic currents in cultured cortical neurons (Tang and Aizenman, 1993). The current study, however, could not directly address this possibility, as we focused only instrinsic excitability independent of synaptic activity. Interestingly, it has been suggested that ketamine’s rapid antidepressant effects may work through simultaneous suppression of T-channels and NMDA receptors in the lateral habenula (Yang et al., 2018). Hence, it is possible that the inhibitory effect of ALA on neuronal NMDA currents could work in concert with inhibition of multiple T-current isoforms in mesolimbic reward system. This remains to be investigated in future studies.

## Conclusion

We have shown now and in our previous study the importance of T-channels in the two largest functional neuronal population in the VTA (DA and GABA neurons) and have examined their role in the transmission of neuronal information. As VTA activity is canonically implicated in reward and motivation, and more recently in pain modulation, we present T-channels as a target worth pursuing in the search for novel treatments for pain and issues regarding drug abuse. Further study of ALA and its impact on neurophysiology is likely to yield more effective therapeutic strategies.

## Data Availability Statement

The datasets generated for this study are available on request to the corresponding author.

## Ethics Statement

The study was approved by the University of Colorado Health Science Center Animal Care and Use Committee, protocol #00159 for the investigator Dr. ST from 02/04/2019-02/04/2022.

## Author Contributions

ST and TW designed the electrophysiological experiments. TW and TS performed electrophysiology experiments and analyses. VJ-T and VT designed the immunohistochemistry experiments. VT performed immunohistochemistry and subsequent imaging. ST and TW drafted the manuscript and all authors participated in revisions. ST and TW were responsible for the overall direction of the project.

## Funding

This study was funded in part by grants from the National Institutes of Health (GRANT# R01GM102525 to ST and R01GM118197 to VJ-T).

## Conflict of Interest

The authors declare that the research was conducted in the absence of any commercial or financial relationships that could be construed as a potential conflict of interest.
