# Supplementary material for: Pharmacological Antagonism of T-Type Calcium Channels Constrains Rebound Burst Firing in Two Distinct Subpopulations of GABA Neurons in the Rat Ventral Tegmental Area: Implications for α-Lipoic Acid
Source: Front Pharmacol. 2019 Nov 26;10:1402. doi: 10.3389/fphar.2019.01402 (PMC6889856; doi:10.3389/fphar.2019.01402)
Supplement: Supplementary file 1 [file DataSheet_1.pdf]

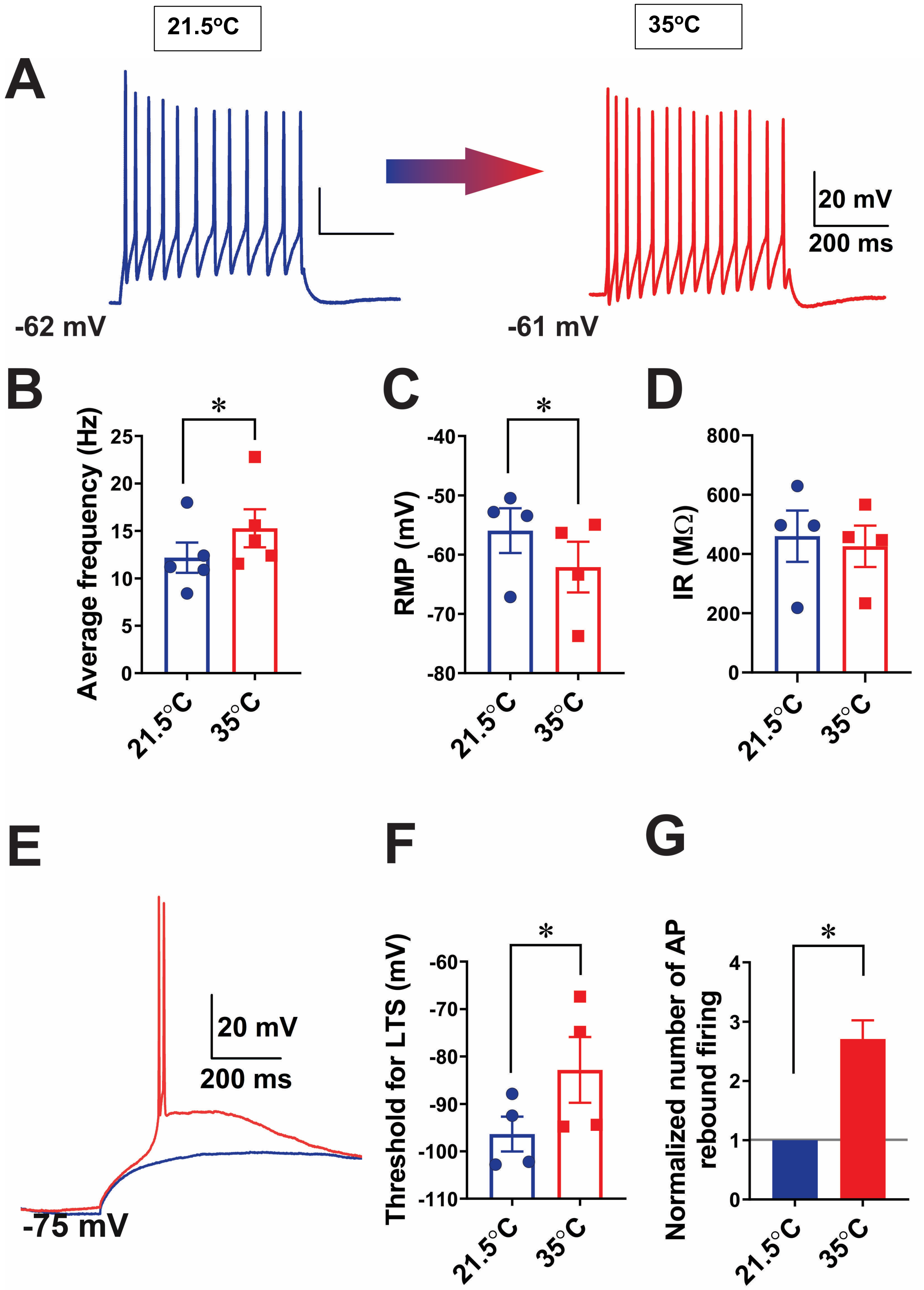

21.5°C
35°C
A
20 mV
200 ms
-62 mV
-61 mV
B
Average frequency (Hz)
C
RMP (mV)
D
IR (MΩ)
E
20 mV
200 ms
-75 mV
F
Threshold for LTS (mV)
G
Normalized number of AP rebound firing

Supplemental Figure S1. Higher temperature increases excitability of VTA neurons in VGAT rats

A, Original traces from a representative VTA neuron recorded at room temperature of 21.5°C (blue trace) and after perfusion with warmer external solution at 35°C (red trace) with a depolarizing current injection of 50 pA. **B**, Switching to the warmer external solution in the same neurons significantly increased averaged cumulative tonic firing frequency for about 20% as determined from the input-output curves (5 neurons from 4 animals; paired two-tailed t-test: $t(4) = 3.19$, $p = 0.033$). **C**, Warmer external solution significantly hyperpolarized the VTA neurons for about 5 mV (4 neurons from 4 animals; paired two-tailed t-test: $t(3) = 3.82$, $p = 0.031$). **D**, Input resistance was not significantly affected after perfusion with warmer external solution. **E**, Original traces from a representative VTA neuron after hyperpolarizing pre-pulse current injection of 50 pA before (blue trace), and after application of warmer external solution (red trace). Note that at room temperature the small current injections elicited only subthreshold depolarization, while the same current injection at warmer temperature elicited a LTCS and rebound burst firing. **F,** The threshold for LTCS was decreased for about 10 mV after perfusion with warmer external solution when compared to the solution at room temperature (4 neurons from 4 animals; paired two-tailed t-test: $t(3) = 4.13$, $p = 0.026$). **G**, Perfusion with warmer external solution increased about 3-fold the average number of cumulative APs in rebound burst firing (4 neurons from 4 animals; one-sample t-test; $t(3) = 5.43$, $p = 0.012$, theoretical mean 1). $^*p < 0.05$

METHODS

VGAT rats were anesthetized briefly with 5% isoflurane and decapitated. Their brains were removed rapidly and placed in a cold (4°C) oxygenated (95 vol % O_2 and 5 vol % CO_2) solution. Live 250- to 300-μm-thick horizontal brain slices were sectioned at 4°C in the same cold solution (in mM): sucrose 260, D-glucose 10, $NaHCO_3$ 26, NaH_2PO_4 1.25, KCl 3, $CaCl_2$ 2, $MgCl_2$ 2, using a vibrating micro slicer (Laica VT 1200S). Brain slices were immediately incubated for 30 min in the following solution (in mM): NaCl 124, D-glucose 10, $NaHCO_3$ 26, NaH_2PO_4 1.25, KCl 4, $CaCl_2$ 2, $MgCl_2$ 2 at 37°C before use in electrophysiology experiments, which were initially done at room temperature. After obtaining baseline values for active and passive membrane properties at room temperature of 21.5°C, warmed external solution at 35°C was perfused for at least 5 minutes and identical excitability protocols were repeated on the same neurons. Temperature of perfusion solution was measured by a thermal probe positioned at the tip of the perfusion chamber. During incubation, slices were constantly perfused with a gas mixture of 95 vol % O_2 and 5 vol % CO_2.

The external solution for current-clamp electrophysiology experiments consisted of the following (in mM): NaCl 125, D-glucose 25, $NaHCO_3$ 25, NaH_2PO_4 1.25, KCl 2.5, $MgCl_2$ 1, $CaCl_2$ 2. The external solution contained the synaptic blockers picrotoxin (20 μM), D-2-amino-5-phosphonovalerate (D-AP5; 50 μM), and 2,3-dihydroxy-6-nitro-7-sulfamoyl-benzo[f]quinoxaline-2,3-dione (NBQX; 5 μM). The internal solution for current-clamp recordings consisted of the following (in mM): potassium-D-gluconate 130, ethylene-glicol-bis(*β*-aminoethylether)*N,N,N',N'*-tetra acetic acid (EGTA) 5, NaCl 4, $CaCl_2$ 0.5, HEPES 10, Mg-ATP 2, Tris-GTP 0.5, pH 7.2. Whole-cell recordings were performed in the VTA neurons visualized under Zeiss optics (Zeiss AXIO Examiner D1, 40x objective). Glass microelectrodes (Sutter Instruments, borosilicate glass with filament OD 1.2 mm) were pulled using a Sutter Instruments P-1000 model and fabricated to maintain an initial resistance of 3-6 MΩ. Neuronal membrane responses were recorded using a Multiclamp 700 B amplifier (Molecular Devices, Foster City, CA, USA). Voltage current commands and digitization of the resulting voltages and currents were performed with Clampex 8.3 software (Molecular Devices) running on a PC-compatible computer. Resulting current traces were analyzed using Clampfit 10.5 (Molecular Devices). Statistical and graphical analyses were performed using GraphPad Prism 7.0 software (GraphPad Software) or Origin 7.0 (OriginLab). Results typically are presented as means ± SEM unless stated otherwise.

Both tonic and burst-firing properties of unidentified VTA neurons were characterized by using multistep protocols that were used to generate input-output curves in VGAT rats. To investigate tonic firing patterns in VTA neurons, we injected a depolarizing current pulse through the recording pipette of 500 ms duration in 10 pA incremental steps starting from 0 pA. To investigate burst-firing patterns, the neurons were injected with hyperpolarizing currents in 25 pA intervals stepping from 0 pA. Subsequent resting membrane potentials (RMP), tonic action potential (AP) frequencies, thresholds to low-threshold calcium spike (LTCS) and input resistances (IR) were determined. RMP was measured at the beginning of each recording and was not corrected for the liquid junction potential.
